# A non-coding insertional mutation of *Grhl2* causes gene over-expression and multiple structural anomalies including cleft palate, spina bifida and encephalocele

**DOI:** 10.1093/hmg/ddad094

**Published:** 2023-06-26

**Authors:** Zoe Crane-Smith, Sandra C P De Castro, Evanthia Nikolopoulou, Paul Wolujewicz, Damian Smedley, Yunping Lei, Emma Mather, Chloe Santos, Mark Hopkinson, Andrew A Pitsillides, Genomics England Research Consortium, Richard H Finnell, M Elisabeth Ross, Andrew J Copp, Nicholas D E Greene

**Affiliations:** Developmental Biology and Cancer Department, Great Ormond Street Institute of Child Health, University College London, London WC1N 1EH, UK; Developmental Biology and Cancer Department, Great Ormond Street Institute of Child Health, University College London, London WC1N 1EH, UK; Developmental Biology and Cancer Department, Great Ormond Street Institute of Child Health, University College London, London WC1N 1EH, UK; Center for Neurogenetics, Brain and Mind Research Institute, Weill Cornell Medicine, New York, New York 10065, USA; William Harvey Research Institute, Barts and the London School of Medicine and Dentistry, Queen Mary University of London, London EC1M 6BQ, UK; Center for Precision Environmental Health, Department of Molecular and Cellular Biology, Baylor College of Medicine, Houston, Texas 77030, USA; Developmental Biology and Cancer Department, Great Ormond Street Institute of Child Health, University College London, London WC1N 1EH, UK; Developmental Biology and Cancer Department, Great Ormond Street Institute of Child Health, University College London, London WC1N 1EH, UK; Department of Comparative Biomedical Sciences, Royal Veterinary College, London NW1 0TU, UK; Department of Comparative Biomedical Sciences, Royal Veterinary College, London NW1 0TU, UK; Genomics England, London E14 5AB, UK; Center for Precision Environmental Health, Department of Molecular and Cellular Biology, Baylor College of Medicine, Houston, Texas 77030, USA; Center for Neurogenetics, Brain and Mind Research Institute, Weill Cornell Medicine, New York, New York 10065, USA; Developmental Biology and Cancer Department, Great Ormond Street Institute of Child Health, University College London, London WC1N 1EH, UK; Developmental Biology and Cancer Department, Great Ormond Street Institute of Child Health, University College London, London WC1N 1EH, UK

## Abstract

Orofacial clefts, including cleft lip and palate (CL/P) and neural tube defects (NTDs) are among the most common congenital anomalies, but knowledge of the genetic basis of these conditions remains incomplete. The extent to which genetic risk factors are shared between CL/P, NTDs and related anomalies is also unclear. While identification of causative genes has largely focused on coding and loss of function mutations, it is hypothesized that regulatory mutations account for a portion of the unidentified heritability. We found that excess expression of *Grainyhead-like 2* (*Grhl2*) causes not only spinal NTDs in *Axial defects* (*Axd*) mice but also multiple additional defects affecting the cranial region. These include orofacial clefts comprising midline cleft lip and palate and abnormalities of the craniofacial bones and frontal and/or basal encephalocele, in which brain tissue herniates through the cranium or into the nasal cavity. To investigate the causative mutation in the *Grhl2^Axd^* strain, whole genome sequencing identified an approximately 4 kb LTR retrotransposon insertion that disrupts the non-coding regulatory region, lying approximately 300 base pairs upstream of the 5’ UTR. This insertion also lies within a predicted long non-coding RNA, oriented on the reverse strand, which like *Grhl2* is over-expressed in *Axd* (*Grhl2^Axd^*) homozygous mutant embryos. Initial analysis of the *GRHL2* upstream region in individuals with NTDs or cleft palate revealed rare or novel variants in a small number of cases. We hypothesize that mutations affecting the regulation of *GRHL2* may contribute to craniofacial anomalies and NTDs in humans.

## Introduction

Orofacial clefts and neural tube defects (NTDs) are among the most common congenital anomalies worldwide. Isolated cleft lip (CL) and cleft lip and palate (CL/P) together affect ~1 per 700 newborns (1,2), while NTDs, such as spina bifida and anencephaly, arise in ~0.5–1 per 1000 pregnancies with much higher rates in some countries (3,4).

Cranial NTDs, such as anencephaly, are typically lethal conditions, while spinal NTDs (spina bifida) and orofacial clefts can be surgically managed. However, this often requires multiple procedures and associated therapy, such that both conditions have lifelong health implications (1,2,5–7). A large number of genes are implicated in the possible causation of CL/P and NTDs on the basis of phenotypes in loss of function models in the mouse. Accumulation of risk variants is thought to contribute to NTDs in humans, but the genetic basis in affected individuals remains largely unknown (8). There has been substantial progress toward determining the genetic basis of orofacial clefting (1,9,10). Genome wide association studies (GWASs), candidate gene analysis and exome sequencing have identified multiple genetic loci, including at least 40 for non-syndromic CL/P, as well as mutations responsible for syndromic forms of CL/P or isolated CP. However, our knowledge of the genetic basis of these disorders remains far from complete (1,7,9,10).

While NTDs and CL/P rarely arise in the same individual, these anomalies have been long recognized to co-occur more frequently than would be predicted by chance (11,12). For example, anencephaly and spina bifida have each been observed at much higher frequency among individuals with CL/P or isolated CP than among the general population (13). Some shared genetic contributors have been identified (14). These observations suggest that some genetic and/or environmental causative factors may be shared. For example, members of the Grainyhead-like (GRHL) family of transcription factors have been implicated in craniofacial anomalies and NTDs in humans and mice (15). Mutations in *GRHL3* contribute to the causation of non-syndromic CL/P as well as syndromic CL/P in Van der Woude syndrome (16–18). Rare mutations in *GRHL3* have also been identified in individuals with spina bifida (19). Consistent with a potential contribution to the NTDs in these individuals, loss, diminished or excess expression of *Grhl3* all cause spina bifida in mice (20–24).

In addition to NTDs, *Grhl3* loss of function also causes a low frequency of CP in mice, associated with abnormal periderm development (16,21). A potential requirement for GRHL2 in craniofacial development is also implied by observations in mice. We and others found that *Grhl2* null embryos exhibit anterior and spinal NTDs but die by E12.5 before lip and palate fusion can be assessed (25). However, an ENU-induced point mutation or diminished craniofacial expression of *Grhl2* causes midline fusion defects and/or infrequent cleft palate (26,27), while knockout in the pharyngeal ectoderm causes occasional micrognathia suggesting a requirement in mandibular and maxillary development (28).

Midfacial development begins at around E9.5 in mice, with formation of the frontonasal process (FNP), the paired maxillary processes (MxP) and mandibular processes (MdP) (29). The FNP subsequently becomes subdivided into the medial (MNP) and lateral nasal (LNP) processes, which are displaced medially as the MxPs grow, allowing the MNPs to meet and fuse in the midline, to give rise to the primary palate at around E11. The paired MxPs appose in the midline and fuse together and with the MNPs, to form the upper lip and primary palate that becomes a continuous structure by E14.5 (30,31). A potential role for *Grhl2* in craniofacial development is consistent with its expression that is already widespread in the surface ectoderm of the FNP by E9.5, as revealed by the lacZ reporter (25) and *in situ* hybridization (32,33).

As in mice with *Grhl2* loss of function, we previously found that spinal NTDs can also be caused by excess expression of *Grhl2* (25) in embryos homozygous for the, as-yet uncharacterized, *Axial Defects* (*Axd*; here denoted *Grhl2^Axd^*) mutation (34). In the current study, we identified previously unrecognized abnormalities in *Grhl2^Axd/Axd^* embryos, including craniofacial anomalies and encephalocele, in which the brain tissues herniate outside the skull. Furthermore, we identified the genomic lesion in *Grhl2^Axd/Axd^* embryos and therefore identified a shared genetic basis for orofacial clefts, encephalocele and spinal NTDs involving an insertional mutation that leads to dysregulation of *Grhl2*.

## Results

### Excess expression of *Grhl2* causes orofacial clefts in mice

**Figure 1:**
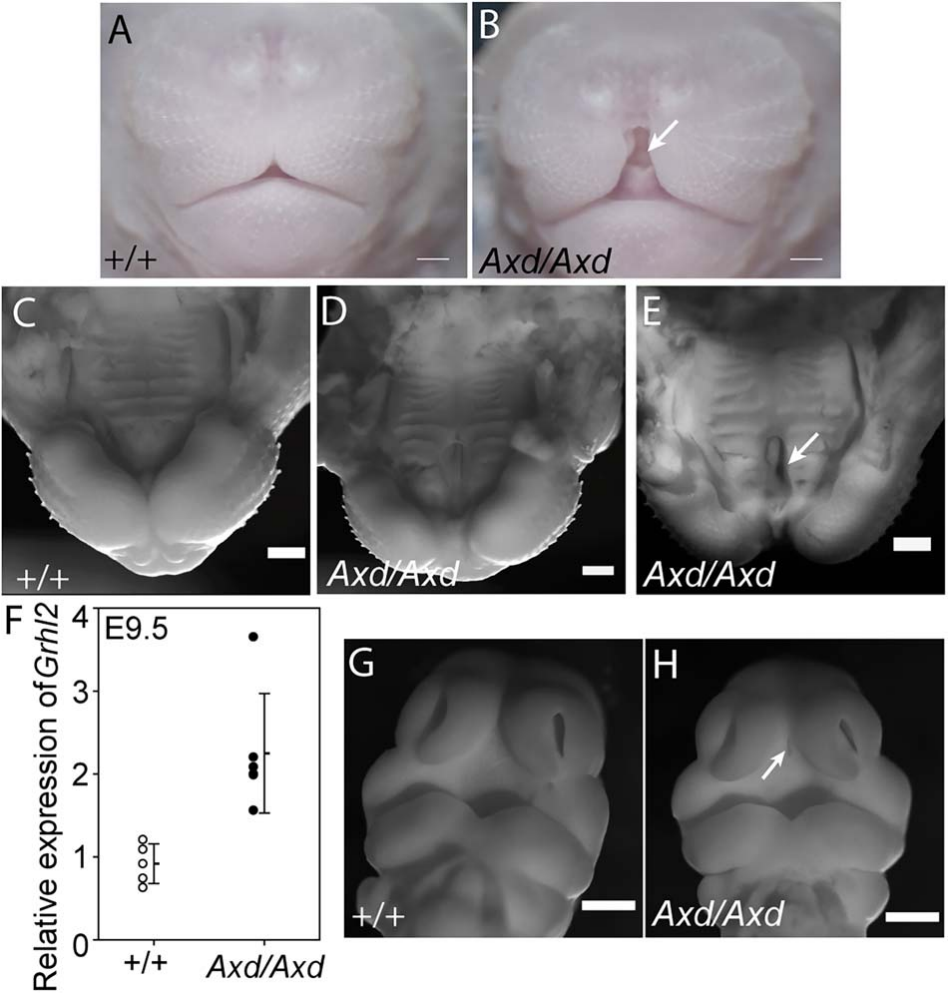
Midline cleft lip and cleft palate in *Grhl2* over-expressing fetuses. Frontal view of snout in wild-type (**A**) and *Grhl2^Axd/Axd^* (**B**) fetus at E18.5 reveals a distinct midline cleft in the homozygous mutant (asterisk in B). (**C**–**E**) View of the lip and palate (lower jaw removed) at E18.5. In comparison with wild-type (C), a midline gap is clearly present in *Grhl2^Axd/Axd^* mutants (asterisks in D,E), with cleft of the primary palate evident in a proportion of embryos (arrow in E). (**F**) *Grhl2* mRNA is significantly more abundant in *Grhl2^Axd/Axd^* embryos than wild-type at E9.5 (^*^P < 0.05, *t*-test). (**G**,**H**) At E11.5, an abnormal midline groove was detectable between the medial nasal prominences of *Grhl2^Axd/Axd^* embryos (arrow in H). Scale bar represents 50 μm (A–E) and 100 μm (G–H).

We previously reported the presence of fully penetrant spina bifida, resulting from failed closure of the spinal neural tube, in *Grhl2^Axd/Axd^* homozygous embryos (25,33). In the current study, we identified additional structural anomalies; examination of late-fetal stages revealed the presence of the midline cleft lip (CL) in homozygous mutants at E17.5–18.5 (*n* = 40, 100%) (Fig. 1A and B). This increased midline gap was clearly visible in whole mount preparations, after removal of the lower jaw (Fig. 1C–E).

*In situ* hybridization analysis previously confirmed that *Grhl2* is over-expressed in the cranial region of *Grhl2^Axd/Axd^* embryos at E9.5, as in the spinal region (33). We confirmed this by qRT-PCR (Fig. 1F), showing that excess abundance of *Grhl2* is present from the beginning of midfacial development. Over-expression of *Grhl2* persisted in the FNP of *Grhl2^Axd/Axd^* embryos at E11.5–12.5 (Supplementary Material, Fig. S1), suggesting the potential for additional abnormalities at later stages of craniofacial development.

**Figure 2:**
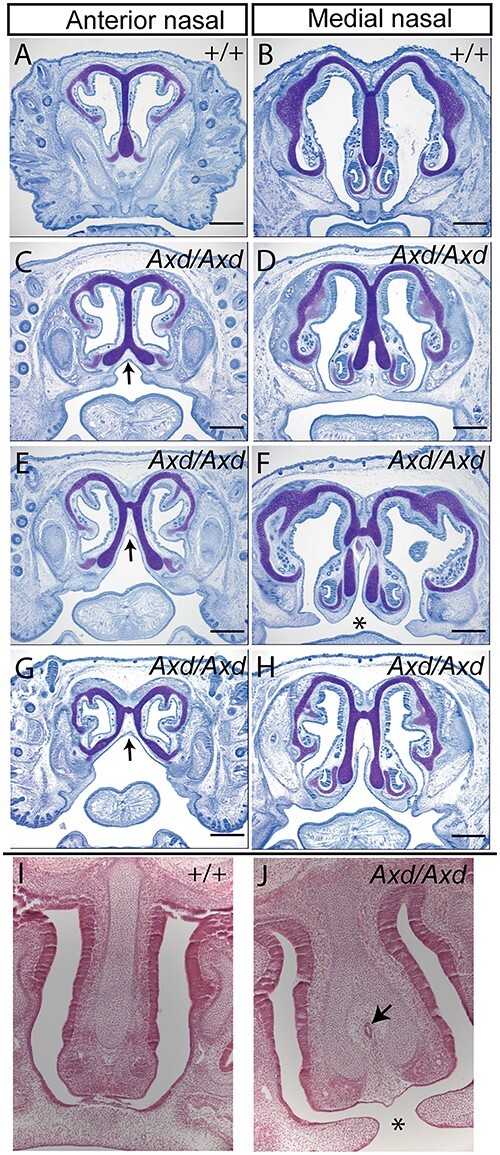
Abnormal nasal septum and cleft palate. (**A**–**H**) Toluidine blue staining of coronal sections showed abnormal splitting of the nasal septum at anterior and medial levels in *Grhl2^Axd/AXd^* fetuses at E17.5. (**I**,**J**) Hematoxylin and eosin–stained sections also revealed splitting of the nasal septum in *Grhl2^Axd/Axd^* (arrows in C,E,G) and the presence of ectopic tissue in the midline (arrow in J). Some fetuses showed failure of fusion of the nasal septum with the primary palate (^*^ in F,J), with small unfused palatal shelves (F,H). Scale bar represents 250 μm.

At E11.5, we noted an abnormal midline groove between the MNPs of *Grhl2^Axd/Axd^* embryos, suggesting a discontinuity in the prospective primary palate (Fig. 1G and H). At later stages, in addition to midline lip, we also observed clefting of the primary palate (Fig. 1E). We confirmed the presence of cleft palate on coronal histological sections, with failed fusion of the palatal shelves apparent in approximately one-third of *Grhl2^Axd/Axd^* fetuses at E17.5 (Fig. 2F and J), but not the secondary palate. In histological sections, it was also apparent that midline CL was accompanied by broadening and/or splitting of the nasal septum (Fig. 2C–H). This phenotype showed variable severity but was present in all fetuses examined (9/9) and was associated with the presence of ectopic midline tissue (Fig. 2J).

### Excess expression of *Grhl2* causes encephalocele in mice

**Figure 3:**
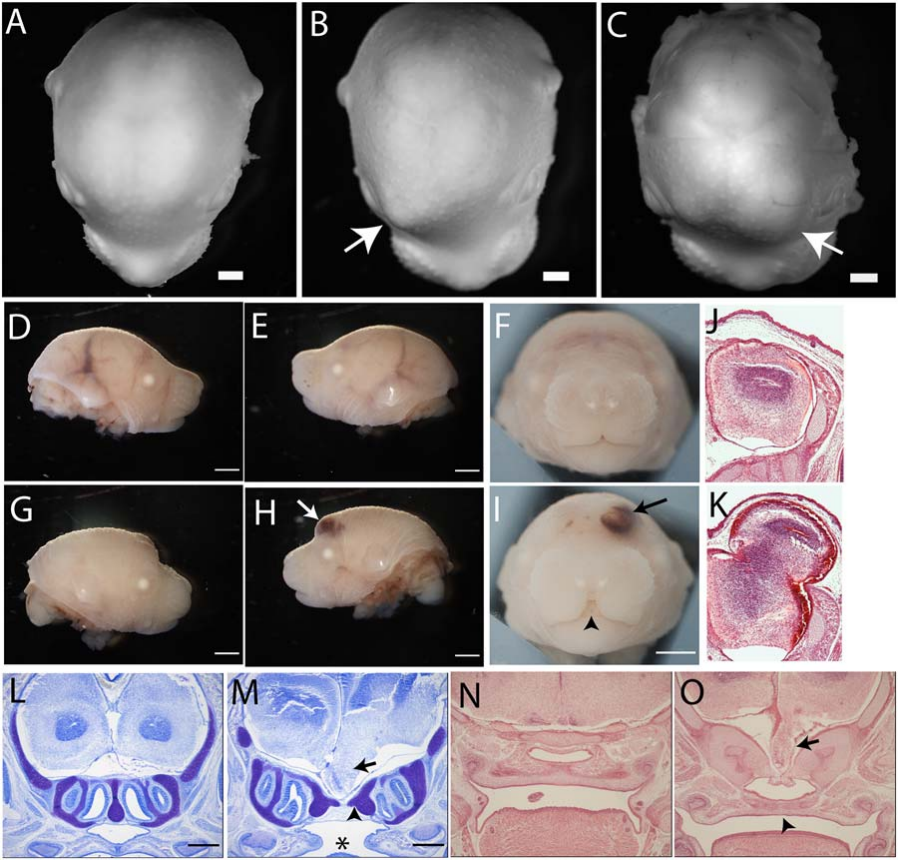
*Grhl2* over-expression causes anterior and basal encephalocele. (**A**–**I**) Examination of fetuses at E18.5 (A–C) and E17.5 (D–I) showed the presence of unilateral bulges in *Grhl2^Axd/Axd^* fetuses that were not present in wild-type (arrow in B,C,H,I) and showed signs of hemorrhage (arrow in H,I). Arrowhead indicates midline cleft lip in I. (**J**,**K**) Hematoxylin and eosin–stained coronal sections at the level of the lesion showed protrusion of skin-covered brain tissue (K), with apparent lateral displacement of the frontal bone (arrow in K). (**L**–**O**) Coronal sections stained with toluidine blue (L,M) or hematoxylin and eosin (N,O) of E17.5 fetuses without anterior encephalocele revealed the presence of abnormal ventral protrusion of brain tissue, at the level of the olfactory bulbs (arrow in M,O). This basal encephalocele corresponded with apparent deflection of the palatine bone (arrowhead in M). Scale bar represents 1 mm.

In addition to midline clefts, we noted a localized frontal bulge on the head, with signs of hemorrhage, in ~35% of *Grhl2^Axd/Axd^* fetuses at E17.5–18.5 (Fig. 3A–I; Table 1). Histological sections showed that this appearance corresponded to herniation of brain tissue outside the forming skull, analogous to human frontal encephalocele (Fig. 3K). These lesions had a characteristic location at the level of the developing frontal bones, were located lateral to the midline and could be on the right or left side. In addition to externally apparent lesions among *Grhl2^Axd/Axd^* fetuses, we observed occasional basal encephalocele in which brain tissue penetrated through the palatine bone and into the nasopharyngeal cavity (with or without cleft palate) (Fig. 3M and O; Table 2). While basal encephalocele was observed in three out of nine fetuses, in other instances, the palatine process appeared to be partially intercepted by brain tissue with an apparent depression at the midline. This abnormality was observed both in isolation or co-occurrent with frontal encephalocele.

**Table 1:** Externally visible abnormalities in *Grhl2* over-expressing fetuses. Craniofacial and spinal phenotypes were noted at E17.5–18.5. Frontal encephalocele was denoted by the appearance of a typically unilateral bulge, usually accompanied by hemorrhage

	Cleft lip	Frontal encephalocele	Spina bifida
*Grhl2^+/+^*	0/45 (0%)	0/45 (0%)	0/45 (0%)
*Grhl2^Axd/Axd^*	40/40 (100%)	15/40 (37.5%)	40/40 (100%)

**Table 2:** Craniofacial abnormalities revealed by histological analysis at E17.5. Coronal section of *Grhl2^Axd/Axd^* fetuses revealed cleft secondary palate and basal encephalocele that were not observed in wild-type littermates

	Cleft palate	Basal encephalocele
*Grhl2^+/+^*	0/9 (0%)	0/9 (0%)
*Grhl2^Axd/Axd^*	5/15 (33%)	3/9 (33%)

### Defects in craniofacial development in *Grhl2* over-expressing mice

**Figure 4:**
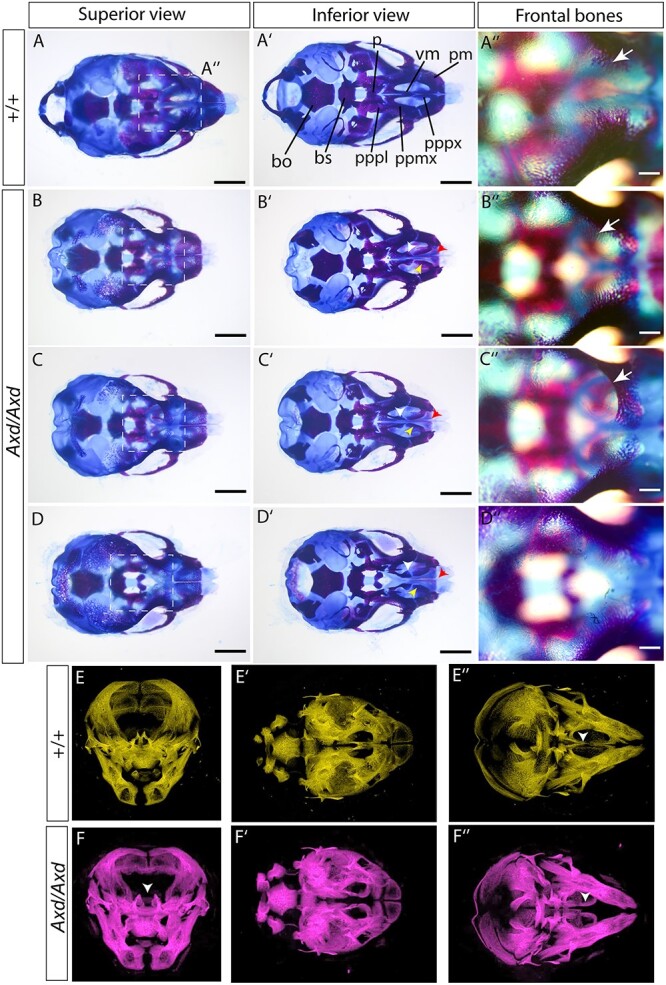
Viscerocranium and frontal bone abnormalities in *Grhl2*^*Axd*/*Axd*^ fetuses. (**A**–**D**) Alcian blue and Alizarin red staining of cartilage and bone was used to stain skeletal elements in the head of wild-type (A) and *Grhl2^Axd/Axd^* (B–D) fetuses. All *Grhl2^Axd/Axd^* fetuses showed a midline cleft lip, and additional anomalies included frontal encephalocele (C″). Images in A″-D″ show enlarged views of the boxed area in A–D. Progress of frontal bone ossification appears to be prevented at the site of encephalocele (white arrow in C″), compared with fetuses without encephalocele (white arrow in A″, B″). Additional abnormalities were noted in the anterior portion of the vomer bone (white arrowheads), posterior portion of the palatine process of the premaxilla (yellow arrowheads), premaxillary bone (red arrowheads) and sometimes in the palatine process of the palatine bone. (**E**,**F**) μCT analysis of E18.5 fetuses confirmed the presence of abnormal midline spacing of palatine bone (white arrowheads; E,F show three orientations of the same fetuses). bo, basioccipital; bs, basisphenoid, p, palatine bone; ppmx, palatine process of maxilla; pppl, palatine process of palatine; pppx, palatal process of premaxilla; pm, premaxilla; vm, vomer. Scale bar represents 1 mm (A–D and A′–D′), 250 μm (A″–D″).

The developmental mechanism underlying encephalocele is not well understood but could potentially involve a defect in the formation of the cranial bones. To investigate this possibility, and to determine whether cleft palate and nasal septum abnormalities are associated with other defects of the viscerocranium, we stained bone and cartilage. As frontal encephalocele was readily identified by E17.5, we conducted Alcian Blue and Alizarin red staining at E18.5 on *Grhl2^+/+^* and *Grhl2^Axd/Axd^* fetuses (Fig. 4A–D). At this stage, the neurocranial bones, including the frontal and ethmoidal bones, are still undergoing ossification in wild-type embryos. In the region of the encephalocele, the progression of ossification of the frontal bone appeared to be impeded (Fig. 4C”, white arrow), compared with wild-type and unaffected mutants (Fig. 4A”, B″ and D″). This timeline would indicate that the protrusion of brain tissue predates the ossification of the affected bones, suggesting that defects in ossification do not underlie the frontal encephalocele.

In addition to the impact on frontal bone ossification and location, a range of defects was observed in other craniofacial bones of *Grhl2^Axd/Axd^* fetuses, particularly within the viscerocranium. In contrast to wild-type fetuses, the anterior portion of the vomer bone was not fused in *Grhl2^Axd/Axd^* (white arrowheads in Fig. 4B’, C′ and D′), nor was the posterior portion of the palatine process of the premaxilla (yellow arrowheads in Fig. 4B’, C′ and D′). Furthermore, the central region of the premaxillary bone appeared to be absent or laterally displaced (red arrowheads in Fig. 4B’, C′ and D′). There was a corresponding increase in the distance between the maxillary processes. Whereas these defects were apparent in all *Grhl2^Axd/Axd^* fetuses, some fetuses exhibited additional abnormalities including the hypoplastic palatine process of the palatine bone (Fig. 4D’).

To complement bone and cartilage staining, we perform micro-computed tomography (μCT) on additional fetuses at E18.5 (Fig. 4E and F; Supplementary Material, Fig. S2; Supplementary  Material, Movie S1–S4). This imaging confirmed the abnormal separation of the premaxilla in the midline in *Grhl2^Axd/Axd^* fetuses (white arrowheads, Fig. 4F and F”).

#### GRHL2 over-expression does not cause craniofacial defects via suppression of GRHL3 function

Mutation of the *GRHL* family member *GRHL3* can cause both non-syndromic and syndromic CL/P, the latter in Van der Woude syndrome (16–18). Similarly, *Grhl3* null mice develop a low frequency of cleft palate, associated with abnormal periderm development, as well as fully penetrant spinal NTDs (16,21). GRHL2 and GRHL3 act as homodimers but can also heterodimerize (35). This raised the possibility that excess GRHL2, in *Grhl2^Axd/Axd^* mutants, could favor formation of heterodimers and thereby inhibit function of GRHL3 leading to malformations that resemble GRHL3 loss-of-function. Arguing against this mechanism, we previously found that increased expression of *Grhl3* (using a *Grhl3*-transgenic line) does not rescue GRHL2-related NTDs. In contrast, moderate over-expression of *Grhl3* and *Grhl2* caused spinal NTDs in compound heterozygous embryos (*Grhl2^Axd/+^*; *Grhl3^+/TgGrhl3^*), even though individual heterozygous embryos develop normally (23). Similarly, in the current study we found that cleft palate can occur among *Grhl2^Axd/+^*; *Grhl3^+/TgGrhl3^* fetuses (one out of two) and *Grhl3^TgGrhl3/TgGrhl3^* over-expressing fetuses (one out of four) at E15.5, showing that increased *Grhl2* is unlikely to cause CL/P via suppression of GRHL3.

### The *Axd* allele corresponds to a retrotransposon insertion in the *Grhl2* promoter

**Figure 5:**
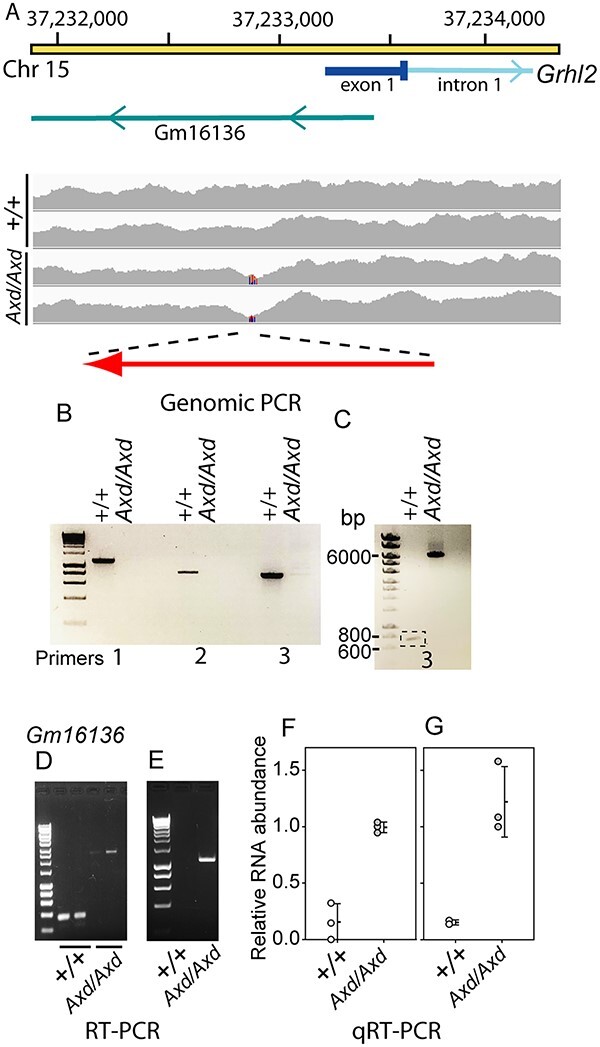
Characterization of the *Axd* mutation. (**A**) Schematic diagram of the region of mouse chromosome 15, which contains the promoter and exon of *Grhl2* and *Gm16136* lncRNA, with WGS coverage in wild-type (*n* = 2) and *Grhl2^Axzd/Axd^* (*n* = 2) samples. Loss of coverage in the *Grhl2^Axd/Axd^* samples is indicated (lines on coverage map) with the corresponding region that was amplified by genomic PCR. Orientation of the inserted IAP transposable element in *Grhl2^Axd^* is indicated (red arrow). (**B**,**C**) Genomic PCR, using primer pairs flanking the region with loss of sequence coverage in mutant embryos, generates products of expected size using wild-type DNA as template but not using *Grhl2^Axd/Axd^* DNA (B), whereas a product of larger size (~6 kb) was amplified using long-range PCR (C) in mutant but not wild-type DNA. (**D**,**E**) RT-PCR using primer pairs that amplify *Gm16136* and (D) flank the insertion site, or (E) include a primer within the inserted sequence, shows that the lncRNA is expressed and has increased size owing to transcription of the inserted sequence in *Grhl2^Axd/Axd^* embryos. (**F**,**G**) Quantitative real-time RT-PCR showed that *Gm16136* has increased expression in *Grhl2^Axd/Axd^* embryos compared with wild-type. The primer pairs amplify isoform (F) 204 or (G) isoforms 202 and 204 (Supplementary Material, Fig. S4).

The *Axd* mutation was previously mapped by linkage analysis to a critical region of 1.1 Mb on mouse chromosome 15 (25). No coding or splice site mutation was identified in *Grhl2* or other genes in this region, yet *Grhl2* was over-expressed in *Axd* mutants and lowering *Grhl2* expression normalized spinal neurulation (25). Here, we further investigated the molecular basis of structural malformations in *Grhl2^Axd/Axd^* embryos by whole genome sequencing (WGS). Within the previously identified *Axd* critical interval, we identified a region with loss of sequence coverage immediately upstream of *Grhl2* in DNA from *Grhl2^Axd/Axd^* but not *Grhl2*^+/+^ embryos (Fig. 5A). Further analysis of reads confirmed loss of homology at Chr15: 37232993 (mouse reference assembly GRCm39), lying 286 bp upstream of the 5’ UTR (exon 1) and 637 bp upstream of the ATG start codon in exon 1. PCR amplification of a region encompassing this site generated products of expected size in wild-type but not in *Grhl2^Axd/Axd^* samples, using genomic DNA as template with several different sets of primer pairs (1–3; Fig. 5B). Using long-range PCR with the same primer pairs, it was possible to amplify a product from *Grhl2^Axd/Axd^* genomic DNA, which was larger than that amplified from wild-type (Fig. 5C). Together, these data demonstrated the presence of an insertion of approximately 4 kb in the 5’ UTR of *Grhl2*. Development of a genotyping method that amplifies wild-type DNA flanking the insertion and an insertion-specific product (using a primer pair that amplifies the genomic DNA-insert transition sequence) confirmed correspondence of the presence of the insertion in homozygous form in all embryos with spina bifida (Supplementary Material, Fig. S3).

To further analyze the insertion, we used the WGS data to interrogate the sequence of mate pair reads of the inserted DNA corresponding to the regions at which chromosome 15 sequence overlaps with the loss of homology. These sequences did not map to a specific genomic locus or chromosome but had homology with sites that were spread across the genome, suggesting that they correspond to repetitive elements. Further sequence coverage of the insertion in a *Grhl2^Axd/Axd^* sample was achieved using a series of overlapping primer pairs designed to ‘walk’ through the insertion, using successive rounds of primer design and Sanger sequencing. Homology searches indicate that the *Axd* insertion corresponds to a retrotransposon of the intracisternal A particle (IAP) family long terminal repeat (LTR) class, with significant sequence homology to regions of DF0004146.1 (IAPEz-Int) and DF0002788.2 (IAPLTR2). This transposable element (TE) is in the reverse orientation with respect to *Grhl2*.

In addition to disruption of the *Grhl2* promoter, the *Axd* insertion lies within the locus for a predicted long non-coding RNA (lncRNA; Gm16136, MGI: 3802137), transcribed from the reverse strand with respect to *Grhl2* (Fig. 5A). The lncRNA has four predicted alternative transcripts (Gm16136–201, −202, −203 and − 204), three of which have an intron with the insertion located in the second of two exons (Supplementary Material, Fig. S4A). Using RT-PCR with samples collected at E10.5, we found that the lncRNA is transcribed in wild-type embryos (Fig. 5D; Supplementary Material, Fig. S4B–E). Using a series of differing primer combinations, we confirmed expression of isoforms 202 and 204, whereas 201 and 203 were not detected in these samples (Supplementary Material, Fig. S4C–E). This is consistent with reported low-level expression of *Gm16136* in RNA-seq data from mouse embryos throughout gestation from E7.5 and postnatally (36,37).

RT-PCR using primer pairs that span the insertion site and within each exon suggests that an expanded lncRNA, containing the inserted sequence, is expressed in *Grhl2^Axd/Axd^* embryos (Fig. 5D and E; Supplementary Material, Fig. S4B, D and E). Moreover, qRT-PCR confirmed that like *Grhl2*, the lncRNA *Gm161136* is expressed at significantly higher levels in *Grhl2^Axd/Axd^* embryos than in wild-type (Fig. 5F and G).

### Analysis of *GRHL2* upstream sequence in human NTDs and OFCs

Identification of multiple structural defects resulting from *Grhl2* over-expression in mice highlights dysregulation of *GRHL2* as a potential contributor to analogous anomalies in humans. As an initial investigation of this possibility, we analyzed the upstream region of *GRHL2* (2.5 kb 5′ upstream of exon 1) using WGS data of individuals with spinal NTDs or cleft palate.

Among 149 individuals with non-syndromic spina bifida (myelomeningocele), we did not detect copy number variants in this region (or within 50 kb). However, we detected six rare or previously unreported single-nucleotide variants (SNVs; Table 3). These include two variants that were not present in the Genome Aggregation Database (gnomAD, build 38, v3.1.2) or 100 000 genomes databases, one of which lies in the 5’ UTR and the other in an LncRNA-encoding gene (ENSG00000289048) on the reverse strand. Another rare variant in this LncRNA (rs551990862, 8: 101492062), with allele frequency of 7.23 e-05 in gnomAD, was found in two individuals with spina bifida but not in 149 ancestry-matched controls. Among a further 132 trios (individual with spina bifida and parents), we identified an additional four rare variants among eight NTD cases (allele frequency of 2 e-4 to 7 e-3 in gnomAD, build 38), none of which were *de novo* mutations (Supplementary Material, Table S1).

**Table 3:** Sequence variants identified in the *GRHL2* upstream region in individuals with NTDs or cleft palate. Genome sequence was screened in a region of 2.5 kb upstream of *GRHL2* (exon 1 is located at chromosome 8: 101492439 (GRCh38 reference sequence). Variants were evaluated for frequency in gnomAD (v3.1.2), in which more than 152 000 high-quality alleles were genotyped for each variant

Chr: Position	Ref > Alt	NTDs	Cleft palate	gnomAD	Additional features/notes
8: 101490371	A > C	1		Absent	
8: 101490607	A > AAAG	1		Absent^a^	
8: 101490786	G > T		1	1 (6.58 e-6)	Micrognathia, Hearing loss
8: 101491044^b^	T > C	2		30 (1.97 e-4)	
8: 101491626^c^	C > T		1	5 (3.29 e-5)	
8: 101492062^c^	A > T	2		11 (7.23 e-5)	
8: 101491563^c^	G > A	1	1	106 (6.97 e-4)	Independent SB & CP cases
8: 101492278^c^	C > CG		1	Absent	Kidney anomaly, Hearing loss
8: 101492438	C > A		1	1 (6.57 e-6)	Micrognathia
8: 101492559^d^	C > .	1		Absent	

^a^Variant not reported but similar variants are present, e.g. A > AAAAAG

^b^Maternally inherited variant (identified in two trios). Inheritance was unknown for the other variants

^c^Located in exon of lncRNA ENSG00000289048

^d^Located in 5’ UTR. Variants except the 5’UTR variant are also present in the intron of lncRNA ENSG00000520268

As a preliminary investigation of the potential contribution of *GRHL2* upstream variants to orofacial clefts, we also screened this region for rare variants (minor allele frequency < 0.1%) in 397 individuals whose annotated clinical phenotype included ‘cleft palate’ from the 100,000 Genomes Project (38). Among 10 high-confidence variants fitting this criterion (Supplementary Material, Table S2), there were 3 variants that were absent in the gnomAD database (v3.1.2), or very rare (only one allele in gnomAD, frequency of 6.6 e-06) (Table 3). We found one known variant in different individuals with cleft palate or spina bifida (Table 3).

*Grhl2* loss of function has been found to cause micrognathia and kidney abnormalities in mice (28,39) and is associated with hearing loss in humans (40,41). We therefore asked whether any of these features co-occurred with cleft palate among the individuals carrying rare SNVs in the *GRHL2* upstream region and found one or more to be present in 5 of the 10 cases (Supplementary Material, Table S3).

## Discussion

*GRHL2* is expressed in multiple epithelia during embryonic development, with loss of function being associated with several abnormalities. In mice, diminished *Grhl2* expression causes cranial and spinal NTDs, split face (25,32,42), abnormal development of maxilla (26), kidneys (39,43), lung (44) and placenta (45), the latter being the likely cause of mid-gestation lethality. In humans, mutations in *GRHL2* have been implicated in autosomal dominant and age-related hearing loss (40,41) and ectodermal dysplasia (with abnormal dentition) (46). While the majority of *GRHL2* mutations identified to date appear to result in diminished function, non-coding intronic variants that increase transcription *in vitro* have been identified in individuals with corneal endothelial dystrophy (47). In addition to a role in craniofacial, neurulation and ectodermal defects, *GRHL2* is thought to play a role in multiple types of cancer as a tumour suppressor or promoter and has predictive prognostic value in breast cancer and colorectal cancers (48).

In mice, we previously found that like loss of expression, *Grhl2* over-expression also causes spinal NTDs (25,33). In the current study, we identified the additional presence of midline cleft lip and palate and abnormalities of the viscerocranium and encephalocele in *Grhl2^Axd^* mice. These findings indicate that both loss and gain of function of *Grhl2* can cause craniofacial abnormalities and spina bifida. We hypothesize that abnormalities of the viscerocranium, affecting, for example, the vomer bone and premaxilla, are an indirect effect of *Grhl2* over-expression arising secondary to midline fusion defects. *Grhl2* is expressed in the epithelia of the nasal processes and in the olfactory epithelium from E10.5–17.5 (25,49). However, it is not expressed in the mesenchyme from which the vomer and premaxilla form by sequential condensation of mesenchyme, chrondogenesis and ossification.

As lip and palate development involve adhesion and fusion of epithelia, as in neural tube closure, it is possible that there are shared features of the underlying molecular and/or cellular mechanisms. GRHL2-related NTDs are accompanied by dysregulation of an epithelial gene signature that includes multiple components of apical junction complexes, such as claudins and *Cdh1* (encoding E-cadherin), and altered integrity of the actomyosin network in the surface ectoderm (SE) of the closing neural folds (33). Like the reciprocal transcriptional changes caused by *Grhl2* under- and over-expression, there is also an opposite effect on actomyosin, with disruption in the *Grhl2*-null SE and an intense actin cable at the SE-neuroepithelium boundary in *Grhl2^Axd/Axd^* embryos. As a result, spinal NTDs are associated with differing morphology and SE biomechanics in *Grhl2* null and over-expressing embryos (33). While loss of cellular adhesion proteins and actomyosin integrity appears to impair the ability of the neural folds to approach one another, and propagate zippering, the over-expression of these molecules in *Grhl2^Axd/Axd^* embryos is hypothesized to prevent SE cell rearrangements required for progression of closure (33). We speculate that a comparable effect on the oral epithelium could similarly prevent tissue events required for lip and palate development, and this should be investigated in future studies.

Alongside NTDs and craniofacial anomalies, we noted the additional presence of encephalocele, a malformation that has not previously been recognized to be caused by dysfunction of GRHL proteins. In humans, encephalocele has an overall prevalence of 1–3 per 10 000 births (50,51). Despite the possibility of surgical repair after birth, the potential damage to brain tissue means that affected individuals may die in infancy or suffer long-term health complications including epilepsy and sensorimotor deficits. However, while encephalocele arises as part of some syndromes in which causative genes have been identified, such as Meckel syndrome (multiple genes) and Knobloch syndrome type 1 (*COL18A1*) (52), in most cases, the genetic basis is not known (53).

Encephaloceles are typically classified by rostro-caudal location with occipital encephalocele being most common (54). Anterior sub-types, such as frontoethmoidal and interfrontal encephalocele, represent a minority of cases in Western Europe and USA but are more frequently represented in South-East Asia (55,56). These types present additional challenges in management owing to the frequently associated craniofacial deformation (57). Anterior encephalocele is rarely found in mouse models, the only well-described examples being *Tet1^Tuft^* (58) and *Apaf1^fog^* (59). Hence, *Grhl2^Axd^* provides a novel mouse model for an encephalocele type that is common in South-East Asian populations and for which the causative mechanisms are almost completely unknown. Similarly, basal encephalocele is a condition which can be associated with midline abnormalities in humans but, to our knowledge, has not previously been described in mouse models.

Encephaloceles are usually categorized as NTDs and are variably described as resulting from failed neural tube closure or post-neurulation abnormalities: two possibilities that are incompatible (51). This question remained controversial, in part owing to a paucity of experimental models (53). In the current study of *Grhl2* over-expressing embryos, spina bifida resulted from incomplete closure of the spinal neural folds whereas the cranial neural tube was always found to be closed by E10.5 and exencephaly (open cranial NTD) was not observed, consistent with previous reports (25,33). Tissue-specific knockout of *Rac1* can lead to either cranial NTDs (with open neural folds) or encephalocele, in which the neural tube is closed (53). Hence, in both *Rac1* and *Grhl2* models, encephalocele definitively arises after neural tube closure is complete.

The genomic mutation underlying *Grhl2* over-expression in the *Axial defects* mouse strain was not previously defined. Here, we identified an insertional mutation, comprising a TE that is located immediately upstream of *Grhl2* exon 1 (and hence within the previously mapped *Axd* critical region) and segregates with the *Axial Defects* phenotype and *Grhl2* over-expression. Analysis of the inserted sequence suggests that the *Grhl2^Axd^* insertional mutation is likely to have resulted from retrotransposition of an active endogenous retroviral element (ERV; also referred to as LTR retrotransposons) of the class II IAP family. While ~10% of the mouse genome consists of ERVs that are mostly inactive or silenced, some ERVs retain retrotransposon activity and it is estimated that ERV insertions account for ~10% of spontaneous mutations in mice (60,61). Among the three major classes of mouse ERV families, the class II IAPs are among the most active group of ERVs and have been associated with numerous insertional mutations observed among spontaneous mutant alleles in mice (60,62). IAP contains transcription regulation motifs including promoter and enhancer elements, and excess or ectopic expression of gene expression driven by IAP LTRs has been well documented among mouse mutant alleles (62,63). In these cases, the TEs are typically oriented with transcription in the opposite direction to the dysregulated gene, with activity of an antisense promoter in the LTR being responsible for ectopic expression (60,62). For example, the genetic basis of the *clf1* mutation was identified as an inserted IAP retrotransposon lying 6.6 kb and in reverse orientation to *Wnt9b* leads to over-expression, which causes CL/P (64). Similarly, transcription in a reverse direction from an IAP TE in the promoter of *Stab2* leads to over-expression in DBA/2 J mice (65). We conclude that activity of an antisense promoter present within the LTR of the *Axd* IAP is the most likely cause of the over-expression of *Grhl2*.

In addition to causing *Grhl2* over-expression, the causative TE insertion also disrupts the *Gm161136* locus, encoding a predicted lncRNA that is expressed at a low level during mouse embryonic development (36,37). We cannot exclude the possibility that *Gm161136* disruption contributes to craniofacial anomalies in the *Axd* strain. However, the expression pattern of *Grhl2* (25,49), and the association of *Grhl2* loss of function with comparable malformations that we observe in *Axd* mutants (midline fusion defects, cleft palate and a brain abnormality that resembles encephalocele (26)), strongly suggest that the anomalies observed in *Axd* mutant embryos result from over-expression of *Grhl2* as shown for spinal neurulation defects in this strain (25). Moreover, there is evidence that *Grhl2* regulates transcriptional targets in the pharyngeal arch epithelium that are also reciprocally regulated in the spinal region of *Grhl2* null and *Axd* mutant embryos (15,33). The function of *Gm161136* is unknown, but we speculate that it may be involved in regulation of *Grhl2* transcription, in which case its disruption could potentially contribute to the over-expression of *Grhl2* in the *Axd* strain caused by the TE insertion.

The effects of loss or gain or function of *Grhl2* in mice suggest it as a compelling candidate gene for analogous anomalies in humans. Limited evidence so far links *GRHL2* and orofacial clefts. GRHL2 loss of function is associated with sensorineural deafness and ectodermal dysplasia (46), the latter of which co-occurs with CL/P in the spectrum of ectrodactyly-ectodermal dysplasia-Cleft Lip/Palate (EEC syndrome) and ankyloblepharon ectodermal defects-cleft lip or palate (AEC syndrome; encompassing Hay–Wells and Rapp–Hodgkin syndromes) (66). *GRHL2* mutations have not been reported in these syndromes, but not all cases have a genetic diagnosis, and causative mutations have been identified in *TP63*, a direct target of *GRHL2* (67). Several studies show that another GRHL2 target, *CDH1*, is also associated with non-syndromic CL/P (68). It is not yet known whether *GRHL2* dysregulation is involved in human NTDs or encephalocele, both of which may co-occur with CL/P, suggesting a possible shared genetic etiology. For example, midline CL/P may co-occur with encephalocele in rare conditions including Meckel syndrome, Sakoda Complex and Morning Glory Syndrome (52,69,70).

Findings in *Grhl2^Axd^* emphasize the potential contribution of regulatory mutations to congenital anomalies. The human genome contains far fewer active ERVs than in mouse, and ERV insertions are thought to contribute a correspondingly lower mutation load. However, other sources of regulatory mutations can contribute to disease. For example, a nucleotide duplication in an enhancer of *IRF6* has been implicated in cleft palate (71).

Our initial analysis of the *GRHL2* promoter region in individuals with spina bifida (the category of NTDs present in *Grhl2^Axd^* mice) or cleft palate revealed several rare SNVs. Among these, four variants were not present in ~76 000 genomes in the gnomAD database and another two were very rare (only one recorded instance in gnomAD). Variants were present in heterozygous form that does not preclude a possible contribution to NTDs or cleft palate, which are thought to result from accumulation of risk variants in many cases. Interestingly, the three individuals with cleft palate that carried the rarest variants also had other features that have been associated with *GRHL2* mutation in humans or mice, including micrognathia, hearing and kidney anomalies (28,39–41). Whether the variants identified in NTDs and cleft palate contribute to these anomalies is as yet unknown and, in the future, functional studies would be required to assess a potential impact of these variants on *GRHL2* regulation.

In summary, we find that *Grhl2* overexpression causes multiple structural malformations in mice, and we propose that *GRHL2* represents a candidate for involvement in human congenital anomalies including NTDs, encephalocele and CL/P.

## Materials and Methods

### Mice and genotyping

Animal studies were carried out under regulations of the Animals (Scientific Procedures) Act 1986 of the UK Government and in accordance with guidance issued by the Medical Research Council, UK in *Responsibility in the Use of Animals for Medical Research* (July 1993) and the National Centre for the 3Rs’ Responsibility in the Use of Animals for Medical Research (2019).

The *Axial defects* (*Axd*) mutation arose spontaneously (34) and is maintained in our laboratory on a BALB/c genetic background (25). Mice and embryos were genotyped by PCR of genomic DNA prepared from ear biopsy or yolk sacs using primers to a closely linked polymorphism as previously described (25). Identification of the *Grhl2^Axd^* insertion mutation allowed PCR genotyping using three primers: 5′ CCAGTTCAGGAAAGGTCGCT and 5′ACTACAGACACTAGGTACCGT, which flank the insertion (and amplify the wild-type allele), with an insert-specific primer 5′ GACTTCAGGTCAACTCCACG.

### Sample collection and tissue processing

Litters were generated by timed matings in which mice were paired overnight, and the day of finding a copulation plug was designated embryonic day 0.5 (E0.5). Embryos and fetuses were dissected between E12.5 and E17.5 and fixed overnight in in 4% paraformaldehyde (PFA) in phosphate-buffered saline (PBS) at 4°C. E17.5–18.5 fetuses were visually inspected for cleft lip and other external anomalies. Whole embryo images were acquired using a Leica stereomicroscope equipped with an IDS UI3080SE camera and uEye software. Scale bars were added to images using Adobe Photoshop CS3. Photographs of frontal views were used to measure the distance between the maxillary regions using the line tool in ImageJ (72).

For production of tissue sections, embryos were washed in PBS, incubated in a series of 25, 50, 75, and 100% ethanols and then placed in fresh 100% ethanol overnight. Embryos were equilibrated in xylene for 1–2 h followed by 50/50 xylene:paraffin wax for 1 h at 60°C. Embryos were subsequently incubated in paraffin wax for 1 h at least four times at 60°C. Subsequently, embryos were positioned and embedded in paraffin wax and then 10–14 μm thick sections were cut.

### H&E staining

Paraffin slides were de-waxed by passage though 100% histoclear 3 × 15 min, followed by 100% ethanol 3 × 5 min and then serial passage through 90, 70, 50 and 30% ethanol for 2 min each. Slides were then washed in H_2_O and stained in hematoxylin for 4 min and then rinsed again in in H_2_O. Slides were then differentiated in acid/alcohol for a few seconds, rinsed in H_2_O and then placed in saturated lithium carbonate for a few seconds. They were then rinsed in H_2_O for at least 5 min and then stained in eosin for 2 min and again rinsed in H_2_O. Slides were then dehydrated by five changes in 100% ethanol and placed in 100% histoclear 3 × 15 min. Lastly, slides were mounted in DPX (Cell Path, SEA-1304-00A) and coverslips were added.

### Toluidine blue staining

Paraffin slides were de-waxed in 100% histoclear for 3 × 10 min and then placed in 100% ethanol for 3 × 5 min and rehydrated by serial passage through 90 and 70% ethanol for 1 min each. Slides were stained in 0.1% toluidine blue in 1% NaCl in H_2_O (pH 2.3) for 5 min and then rinsed for 3 × 1 min in H_2_O. Slides were then dehydrated in 70% followed by 90% ethanol for 1 min each and then incubated for 3 × 5 min in histoclear. Lastly, slides were mounted in DPX and coverslips were added.

### Alcian blue/alizarin red bone staining

Skeletal preparations from E18.5 fetuses were concurrently stained with Alizarin Red and Alcian Blue (73,74).

### μCT

Fetuses were fixed in ice-cold 4% PFA, washed and stored in PBS. Prior to scanning, the head was removed and dehydrated to 70% ethanol. Samples were scanned using a Skyscan 1172F μCT scanner (Bruker), using a 0.5 mm aluminum filter, 5 μm pixel size and an x-ray source operated at 40 kV voltage and 250 μA energy. The 2D X-ray projection images were reconstructed into tomograms using NRecon software (Bruker). The same settings were applied across all the samples. Images were then processed in FIJI (75) for slice removal and cropping to reduce file size. 3D movies and 2D snapshots were made with IMARIS Microscopy Image Analysis software. Colors were artificially added, and some background correction was made by adjusting brightness and contrast.

### Next-generation sequencing

*Ghrl2^Axd/Axd^* and *Grhl2*^+/+^ DNA was extracted from embryonic tissue using lysis buffer (100 mM Tris pH 8.5; 5 mM EDTA; 0.2% SDS; 200 mM NaCl) and 10 *μg/mL proteinase K incubated overnight at* 55°C. DNA was subsequently cleaned up using the QIAquick PCR purification kit (Qiagen, #28104) as per manufacturer’s instructions. Samples were then sent for sequencing on a NovaSeq 6000 platform to ~400 million 150 bp paired-end reads per sample. Subsequently reads were trimmed using cutadapt paired-end trimming and then aligned to Genome Reference Consortium Mouse Build 38 (mm10) using a bowtie2 paired-end aligner.

### PCR, long-range PCR and sequencing

The region encompassing the putative *Grhl2^Axd^* insertion was amplified by standard or long-range PCR using flanking primers (Primer set 3, Supplementary Material, Table S1). PCR bands were purified and sequenced (*n* = 3 *Grhl2^Axd/Axd^* and 1 *Grhl2*^+/+^). Initial sequencing used the flanking primers, and the insertion was further characterized by additional sequencing using a *Grhl2^Axd/Axd^* genomic DNA sample and a series of primers designed through the insert (Supplementary Material, Table S3). Homology searches for transposable elements were conducted using the Dfam database (https://dfam.org/home).

### RT-PCR and quantitative real-time RT-PCR

For analysis of *Grhl2*, samples comprised the cranial region cut at the level of the first branchial arch in embryos at E9.5 (stage-matched at 17–19 somites; *n* = 6 per genotype). Analysis of the *Gm161136* lncRNA was performed using RNA prepared from whole embryos at E10.5. See Supplementary Material, Table S3 and Supplementary Material, Figure S4 for details of primer sequences.

### WGS analysis of human SB cases

Case–control analysis using WGS data included 149 human case subjects with non-syndromic spina bifida that displayed myelomeningocele, as well as 149 ancestry-matched controls. We also analyzed WGS data from a further 132 trios, comprising individual with spina bifida and parent. DNA samples were sequenced (Illumina HiSeq 2500) yielding short insert paired-end reads of 2 × 100 base pairs (bp). Case–control pairing based on genomic ancestries and single nucleotide variant calling were conducted as previously described (76). In brief, the PaM algorithm used the genetic distances of nine admixture proportions to provide optimal case–control pairs as well as ensuring an ancestry balanced cohort. Genome Analysis Tool Kit (GATK v4) was deployed for variant calling and joint genotyping. SNVs and InDels that passed quality control metrics were further annotated using Variant Effect Predictor (VEP v.95). For copy number variant (CNV) calling and annotations, high-confidence custom pipelines were deployed as previously detailed (77). Variants were considered rare if they occurred with less than 1% minor allele frequency according to population genetic databases (gnomAD, DGV). Visual validation and inspection of genomic variants was conducted using Integrative Genomics Viewer (IGV).

### Variant analysis in cleft palate

BCFtools was used to query an aggregated VCF available in the Genomics England research environment. This VCF was generated from 78 195 WGS sequences from the 100,000 Genomes Project. All variants with a minor allele frequency < 0.1% in this cohort in the upstream region were extracted and then the presence, or not, of clefting in the associated sample was annotated. The latter was based on the Human Phenotype Ontology term annotations of the samples for oral cleft (HP:0000202) or any descendant terms in the ontology.

## Supplementary Material

Supplementary_Materials_Tables_ddad094

Supplementary_Material_Figures_to_submit_ddad094

## Data Availability

Data is available from the authors upon request.
