# Supplementary material for: A non-coding insertional mutation of Grhl2 causes gene over-expression and multiple structural anomalies including cleft palate, spina bifida and encephalocele
Source: Hum Mol Genet. 2023 Jun 26;32(17):2681–92. doi: 10.1093/hmg/ddad094 (PMC10460492; doi:10.1093/hmg/ddad094)
Supplement: Supplementary_Materials_Tables_ddad094 [file supplementary_materials_tables_ddad094.docx]

**Supplementary Table 1. Rare single nucleotide variants identified in individuals with NTDs**

| **NTDs** |  |  |  |  |
| --- | --- | --- | --- | --- |
| **Chr: Position** | **Ref > Alt** | **No. NTDs** | **gnomAD** | **Inheritance** |
| 8: 101,490,371 | A > C | 1 | Absent | unknown |
| 8: 101,490,582 | CAAAAAA > C | 1 | 515 (6.38e-3) | Paternal |
| 8: 101,490,607 | A > AAAG | 1 | Absent^1^ | unknown |
| 8: 101,491,044 | T > C | 2 | 30 (1.97e-4) | Maternal |
| 8: 101,491,120 | A > G | 4 | 658 (4.32e-3) | Paternal (2), Maternal (2) |
| 8: 101,491,563^2^ | G > A | 1 | 106 (6.97e-4) | unknown |
| 8: 101,492,062^3^ | A > T | 2 | 11 (7.23e-5) | unknown |
| 8: 101,492,075^3^ | T > C | 2 | 1018 (6.69e-3) | Paternal, unknown |
| 8: 101,492,559^4^ | C > . | 1 | Absent | unknown |

NTDs comprised 149 individuals with non-syndromic spina bifida and 149 ancestry-matched controls, together with 132 trios (comprising affected individual and both parents), in which inheritance could be determined. ^1^ This variant was not present in gnomAD but similar variants were present e.g. AAAAAG. ^2^ Variant identified in an individual with cleft palate and another with spina bifida. ^3^ Variant located in exon of lncRNA ENSG00000289048, which encodes two transcripts ENST00000701971 (exon at 101,491,259 - 101,492,302) and ENST00000690034 (two exons at 101,491,631 – 101,491,819 and 101,491,820 – 101,492,298). ^4^ Variant located in 5’ UTR. All variants except the 5’UTR variant are also present in the intron of lncRNA ENSG00000520268.

**Supplementary Table 2. Rare single nucleotide variants identified in individuals with cleft palate**

| **Cleft palate** |  |  |  |  |
| --- | --- | --- | --- | --- |
| **Chr: Position** | **Ref > Alt** | **Samples with cleft** | **Total no. (%) in 78,195 samples** | **gnomAD (number & frequency)** |
| 8: 101,490,404 | G > A | 1 | 13 (0.0166) | 135 (8.91e-4) |
| 8: 101,490,786 | G > T | 1^a, b^ | 2 (0.0026) | 1 (6.58e-6) |
| 8: 101,491,177 | T > C | 1 | 41 (0.0524) | 18 (1.18e-4) |
| 8: 101,491,239 | C > T | 1 | 82 (0.1049) | 20 (1.32e-4) |
| 8: 101,491,563^1^ | G > A | 1 | 76 (0.0972) | 106 (6.97e-4) |
| 8: 101,491,610^2^ | A > G | 1 ^c^ | 57 (0.0729) | 16 (1.05e-4) |
| 8: 101,491,626^2^ | C > T | 1 | 7 (0.0090) | 5 (3.29e-5) |
| 8: 101,492,278^2,3^ | C > CG | 1 ^b, c^ | 5 (0.0064) | Not found |
| 8: 101,492,363 | G > A | 1 ^a^ | 54 (0.0691) | 37 (2.43e-4) |
| 8: 101,492,438^4^ | C > A | 1 ^a^ | 15 (0.0192) | 1 (6.57e-6) |

Cleft palate comprised 397 individuals and the number (and percentage) of alleles within 78,195 genotyped samples in the 100,000 genomes database is indicated. Additional phenotypes that correlate with *GRHL2* associated abnormalities in human or mouse studies are indicated. The data from gnomAD (v3.1.2) indicates number of alleles and frequency among at least 152,500 high quality alleles genotyped. ^1^ Variant identified in an individual with cleft palate and another with spina bifida. ^2^ Variant located in exon of lncRNA ENSG00000289048, which encodes two transcripts ENST00000701971 (exon at 101,491,259 - 101,492,302) and ENST00000690034 (two exons at 101,491,631 – 101,491,819 and 101,491,820 – 101,492,298). ^3^ A different variant has been reported at this position; C > T annotated in dbSNP as rs1407210757. ^4^ Variant located immediately adjacent to 5’ UTR. All variants except the 5’UTR variant are also present in the intron of lncRNA ENSG00000520268. Co-occurring abnormalities included ^a^ micrognathia, ^b^ hearing loss, and ^c^ kidney abnormalities.

**Supplementary Table 3 – Primers used for genomic PCR, RT-PCR and sequencing**

| **Primer set** | **Forward** | **Reverse** | **Comment** |
| --- | --- | --- | --- |
| **Genomic PCR** | |  |  |
| **1** | 5’-TACTTCGCACTGTTCCGGAC | 5’-GAGATGGCAGAACAAGAGCTA | Fig. 5B |
| **2** | 5’-TGTTCCGGACCAGAAGAGAAC | 5’-GAGATGGCAGAACAAGAGCTA | Fig. 5B |
| **3** | 5’ GGCCACCTTAAGAGTCTATTC | 5’ GATCGCTGCAATGGGCAGACA | Fig. 5B,C |
|  |  |  |  |
| **RT-PCR** |  |  |  |
| **4** | 5’-CACTAAAGGGTACAAGCCCGA | 5’-TGGTGAACTGGCCATTTGGT | Fig. 5D |
| **5** | 5’-GACTTCAGGTCAACTCCACG | 5’-TGGTGAACTGGCCATTTGGT | Fig. 5E |
| **6** | 5’-CACTAAAGGGTACAAGCCCGA | 5’- ACGGTACCTAGTGTCTGTAGT | Fig. 5F |
| **7** | 5’- TGTGGGAGTTCTGACAGGGA | 5’-CGGCACTAGGTTTGGTTGTT | Fig. 5G |
| **8** | 5’- CCCGAGATGGCAGAACAAGA | 5’-TGGTGAACTGGCCATTTGGT | Fig. S3C |
|  |  |  |  |
| **Sequencing primers** | |  |  |
| **F2** | 5’-TGTTCCGGACCAGAAGAGAAC |  |  |
| **InsF1** | 5’ CGTGGAGTTGACCTGAAGTC |  |  |
| **InsF2** | 5’ AAGCACTCCATGCCTCAAGAC |  |  |
