# Supplementary material for: A non-coding insertional mutation of Grhl2 causes gene over-expression and multiple structural anomalies including cleft palate, spina bifida and encephalocele
Source: Hum Mol Genet. 2023 Jun 26;32(17):2681–92. doi: 10.1093/hmg/ddad094 (PMC10460492; doi:10.1093/hmg/ddad094)
Supplement: Supplementary_Material_Figures_to_submit_ddad094 [file supplementary_material_figures_to_submit_ddad094.pdf]

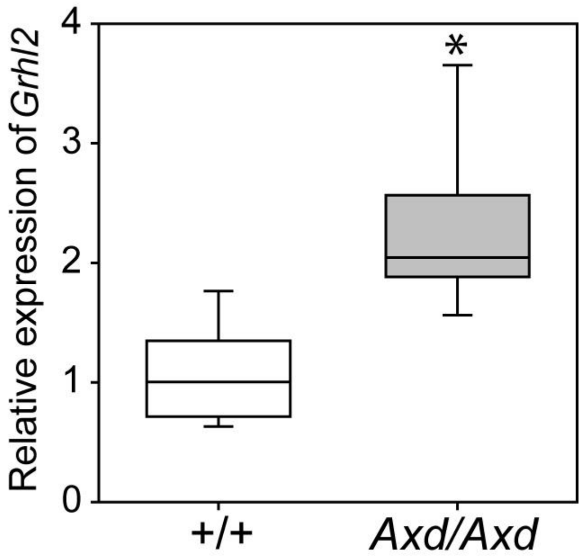

Supplementary Figure 1. Expression of *Grhl2* in the cranial region at E11.5. The frontonasal process was micro-dissected at E11.5 for preparation of RNA samples and qRT-PCR (n = 4 per genotype). *Grhl2* mRNA was significantly more abundant in *Grhl2*$^{Axd/Axd}$ embryos than in wild-type littermates (*, significant difference compared with +/+; $p<0.01$).

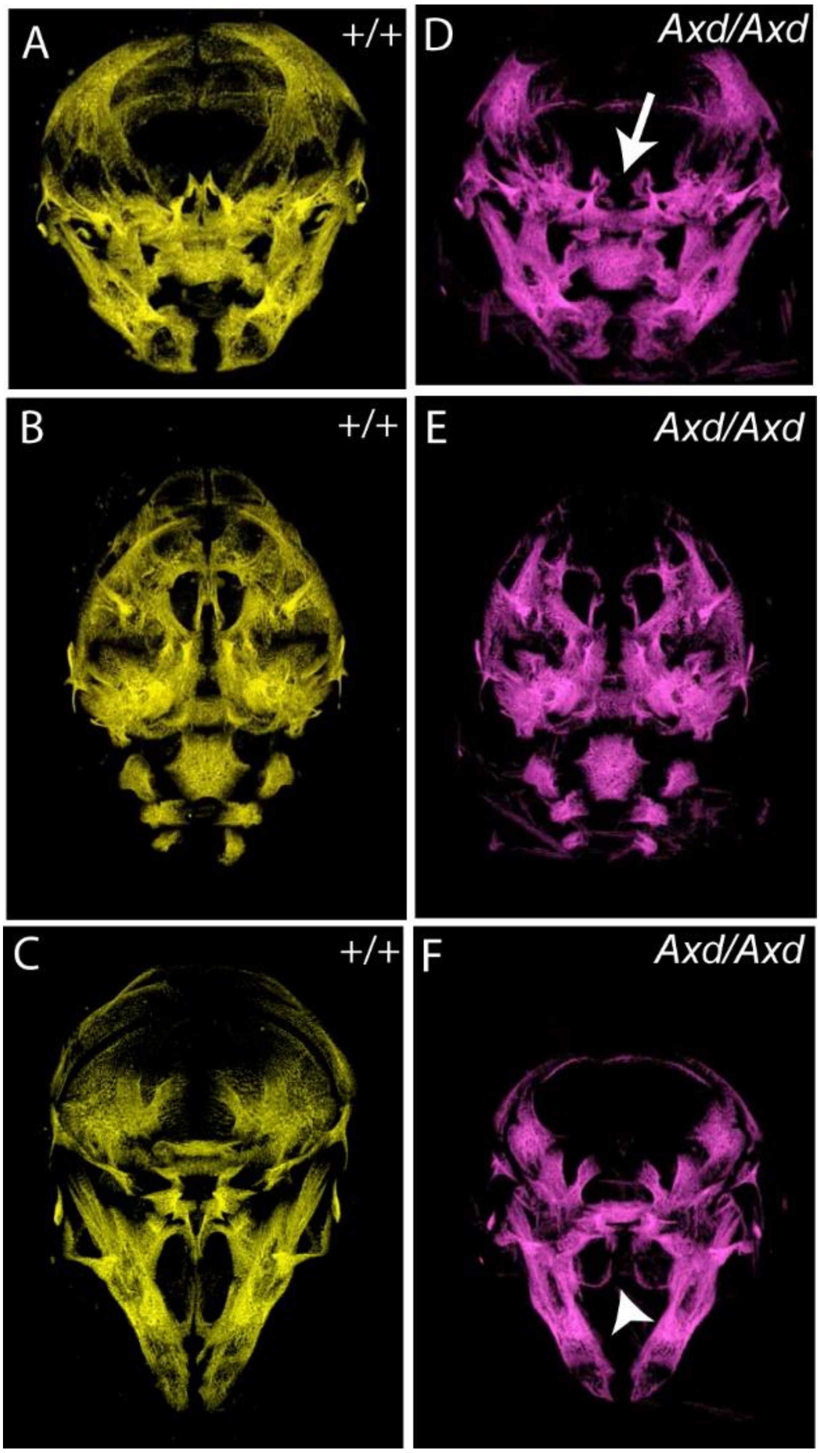

Supplementary Figure 2. MicroCT analysis of E18.5 embryos. Analysis of fetuses at E18.5 (examples are distinct from those shown in Figure 4). Abnormal midline spacing of the palatine bones (arrowhead in D and F) is visible in *Grhl2*$^{Axd/Axd}$ embryos compared with wild-type. Figures show three orientations of each skull (A-C and D-F).

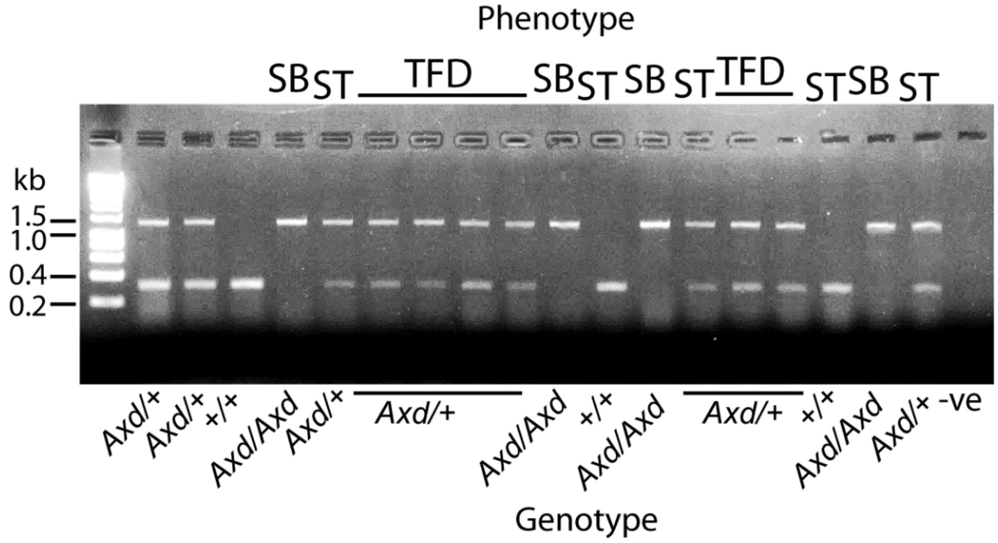

Supplementary Figure 3. Genotyping of *Grhl2*^{Axd} mice based on presence of genomic insertion. A multiplex PCR genotyping method using 3 primers was established using known heterozygous and wild-type genomic DNA, in which the *Grhl2*^{Axd} allele generates a product of higher molecular weight (lanes 1-3). Among a series of fetuses collected at E17.5 the caudal phenotypes were classified as unaffected with straight tail (ST), tail flexion defect (TFD) or spina bifida (SB). Embryos genotyping as *Grhl2*$^{+/+}$ all exhibit ST, while all embryos that genotype as homozygous for the *Grhl2*$^{Axd/Axd}$ insertion exhibited spina bifida. *Grhl2*$^{Axd/+}$ heterozygous embryos frequently exhibit tail flexion defects as predicted from previous studies.

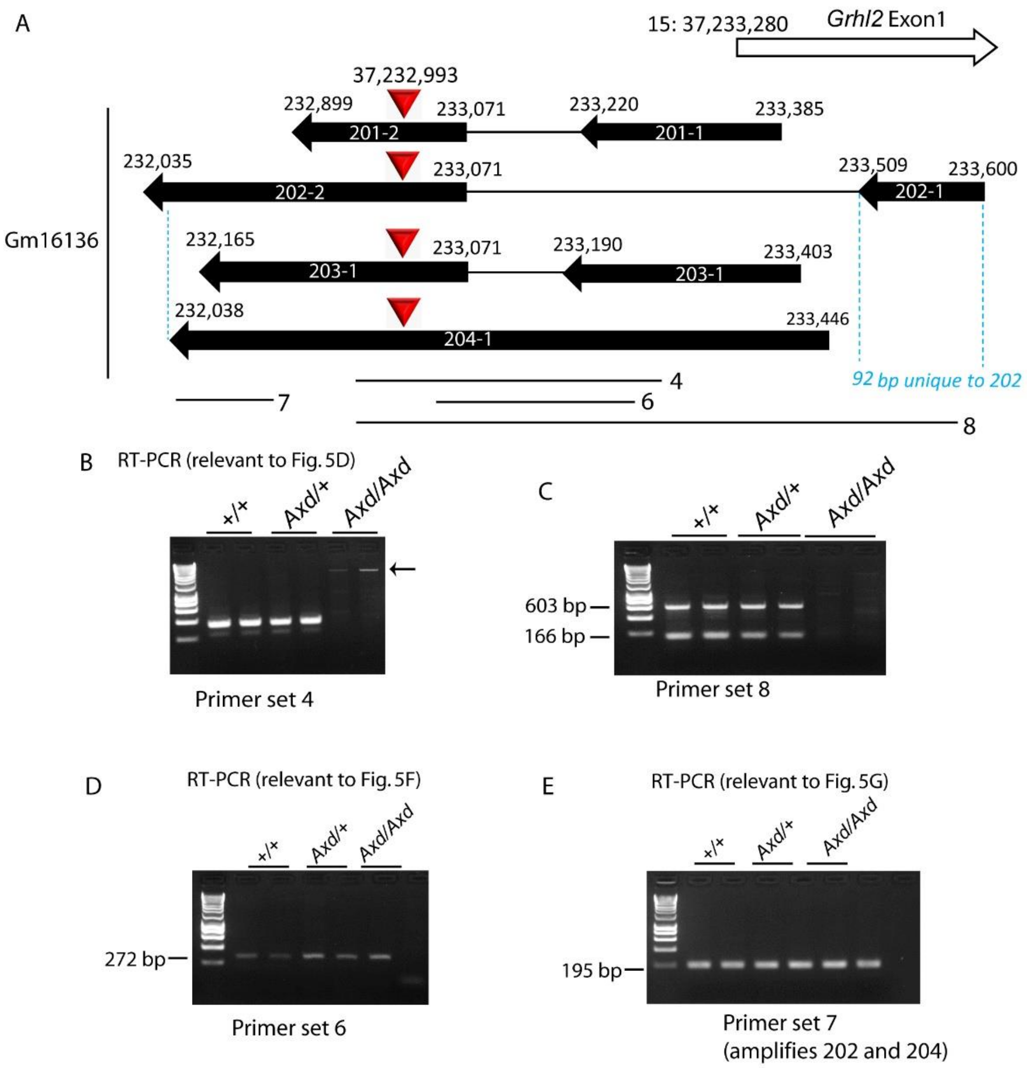

Supplementary Figure 4. Structure and expression of Gm16136 lncRNA in mouse embryos. (A) Gm136 has 4 annotated isoforms with the locations indicated (mouse reference assembly GRCm39) on the reverse strand to *Grhl2* (position of exon 1 on the forward strand is indicated), with the position of the *Axd* insertion indicated by red arrowhead. Lines indicate the predicted products generated using the numbered PCR primer pairs. (B) RT-PCR using primers that flank the *Axd* insertion (and could amplify 201, 203, and 204 isoforms) indicate expression of the lncRNA in wild-type and heterozygous embryos and expression of a larger RNA (with insert; arrow) in $Grhl2^{Axd/Axd}$ embryos. (C) Specific primers (set 8) to the annotated 202 isoform indicate that this isoform is expressed in spliced and unspliced forms, corresponding to the 166 and 603 bp band, respectively. (D) qRT-PCR products using primer set 6 which amplifies a single band corresponding to isoform 204. (E) qRT-PCR products using primer set 7 which can amplify isoform 202 and 204. Amplification of a product from $Grhl2^{Axd/Axd}$ embryos confirms expression of the 3' part of the lncRNA after the *Axd* insertion.
